# ICTV Virus Taxonomy Profile: *Portogloboviridae*

**DOI:** 10.1099/jgv.0.001605

**Published:** 2021-06-04

**Authors:** David Prangishvili, Ying Liu, Mart Krupovic, ICTV Report Consortium

**Affiliations:** ^1^​ Institut Pasteur, 25, Rue du Dr Roux, 75015 Paris, France; ^2^​ Ivane Javakhishvili Tbilisi State University, Tbilisi 1079, Georgia; ^3^​ Archaeal Virology Unit, Institut Pasteur, 75015 Paris, France

**Keywords:** ICTV, taxonomy, *Portogloboviridae*

## Abstract

*Portogloboviridae* is a family of viruses with circular, double-stranded DNA genomes of about 20 kbp. Their icosahedral virions have a diameter of 87 nm, and consist of an outer protein shell, an inner lipid layer and a nucleoprotein core wound up into a spherical coil. Portogloboviruses infect hyperthermophilic archaea of the genus *Saccharolobus*, order Sulfolobales and are presumably nonlytic. Portogloboviruses encode mini-CRISPR arrays which they use to compete against other co-infecting viruses. This is a summary of the International Committee on Taxonomy of Viruses (ICTV) Report on the family *Portogloboviridae*, which is available at ictv.global/report/portogloboviridae.

## Virion

**Fig. 1.**
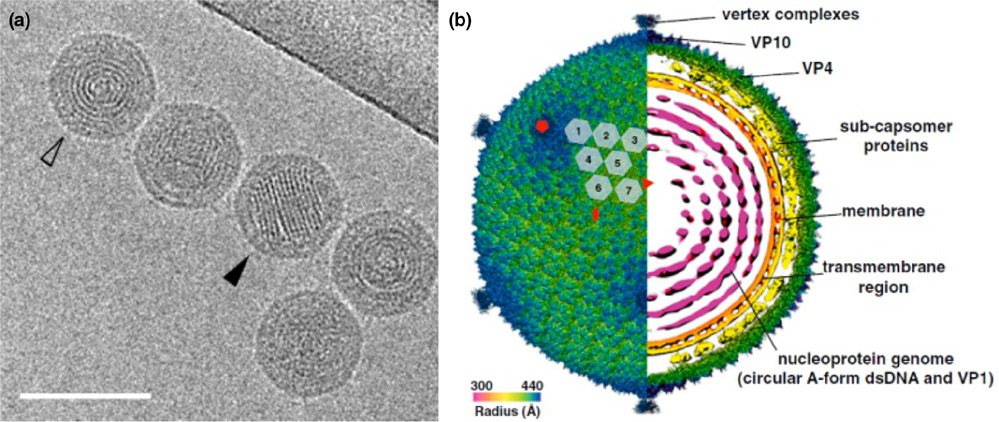
Electron micrograph of Sulfolobus polyhedral virus 1 virions. (**a**) Sample embedded in vitreous ice. Open arrowhead - projection in axial view, filled arrowhead - projection in side view. Scale bars, 100 nm [from [1], copyright American Society for Microbiology]. (b) Overall cryo-EM density map of Sulfolobus polyhedral virus 1, rendered to display the outer capsid (left half) and a central slice showing the interior of the virus (right half). The virus is coloured by radius. The red rectangle, triangle and pentagon denote the twofold, threefold and fivefold axes of symmetry, respectively. The grey numbered hexagons outline the hexameric VP4 capsomers from one asymmetric unit of the icosahedral capsid [2].

Virions are icosahedral, with a diameter of about 87 nm from vertex to vertex and 83 nm from facet to facet (Table 1, Fig. 1a) and consist of three structural units: (a) the outer icosahedral protein shell, (b) the subcapsomer proteins and lipid membrane and (c) a circular nucleoprotein wrapped into a spherical core (Fig. 1b) [1, 2]. The outer shell carries capsid proteins VP4 and VP10, both displaying a single jelly-roll fold. VP10 and VP4 form pentameric and hexameric capsomers, respectively, which assemble into a *T*=43 icosahedral shell. The fivefold vertices of the capsid are decorated with the vertex complexes extending above the capsid surface and potentially involved in receptor binding [2]. The internal membrane of the virus also displays an icosahedral shape, presumably due to the presence of an intricate array of subcapsomer proteins that span the space between the membrane and the external icosahedral capsid [2, 3]. Lipids are selectively acquired from the pool of host lipids, with the lipid compositions in the viral and host membranes being quantitatively very different [2]. The nucleoprotein filament, formed by multiple copies of the nucleocapsid protein VP1 and circular dsDNA, is condensed into a globular core with a characteristic appearance of concentric rings, seen in the axial view, and striations, seen in the side view (Fig. 1a) [1, 2]. The viral dsDNA is stored in the capsid in the A-form (11.0 bp/turn) [2], as previously observed for filamentous archaeal viruses of the realm *Adnaviria* [3, 4].

**Table 1.** Characteristics of members of the family *Portogloboviridae*

Example:	Sulfolobus polyhedral virus 1 (KY780159), species *Sulfolobus alphaportoglobovirus 1,* genus *Alphaportoglobovirus*
Virion	Icosahedral virion, 87 nm in diameter, consisting of an outer protein shell, an inner lipid layer and a spherical nucleoprotein core
Genome	Circular, double-stranded DNA genome of about 20 kbp
Replication	Viral replication occurs without observable cell lysis
Translation	Not characterized
Host range	Hyperthermophilic archaea of the genus *Saccharolobus*, order Sulfolobales
Taxonomy	One genus, two species

## Genome

**Fig. 2.**
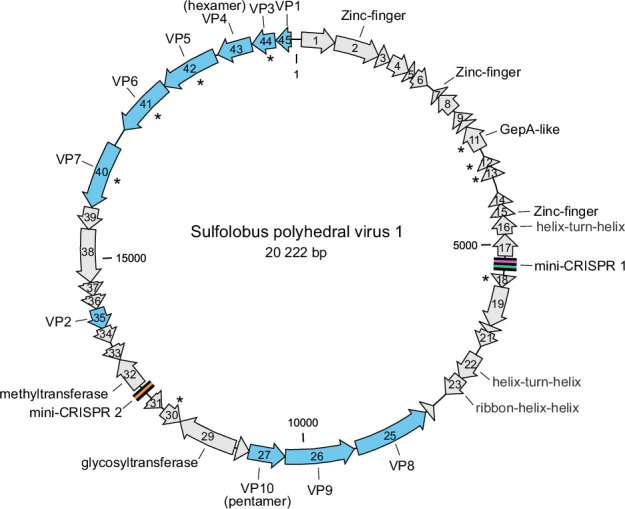
Genome map of Sulfolobus polyhedral virus 1. Genes encoding virion structural proteins are shown in blue; those encoding predicted membrane proteins are indicated with asterisks.

The circular dsDNA genome of Sulfolobus polyhedral virus 1 is 20 222 bp long and contains 45 open reading frames (ORFs) [1, 5]. ORFs are generally short (median 103 codons) and are tightly arranged, occupying 89.1 % of the genome (Fig. 2). Sequence analyses allow the assignment of putative functions to nine ORFs, of which seven encode putative DNA-binding proteins and the other two encode the glycosyltransferase and the methyltransferase (Fig. 2) [1].

## Replication

Viral replication occurs by chronic infection without an apparent lytic cycle [1]. No information is available on entry or egress. The viruses do not encode identifiable DNA and RNA polymerases or other recognizable genome replication initiation proteins [6] and, as in the case of most other viruses that infect members of the Sulfolobales, apparently depend on the host machinery for genome replication and transcription [7]. Portogloboviruses carry mini-CRISPR arrays containing spacers targeting each other as well as other viruses, exemplifying a mechanism promoting inter-viral conflicts and superinfection exclusion in extreme environments [8].

## Taxonomy

Current taxonomy: www.ictv.global/taxonomy. The family *Portogloboviridae* comprises a single genus, *Alphaportoglobovirus*, with two species. Portogloboviruses infect members of the hyperthermophilic archaeal genus *Saccharolobus*, order Sulfolobales.

## Resources

Full ICTV Report on the family *Portogloboviridae*: www.ictv.global/report/portogloboviridae.
